# Systematic review of pragmatic randomised control trials assessing the effectiveness of professional pharmacy services in community pharmacies

**DOI:** 10.1186/s12913-021-06150-8

**Published:** 2021-02-17

**Authors:** R. Varas-Doval, L. Saéz-Benito, M. A. Gastelurrutia, S. I. Benrimoj, V. Garcia-Cardenas, F. Martinez-Martínez

**Affiliations:** 1 Spanish General Pharmaceutical Council, Villanueva 11, 28001 Madrid, Spain; 2 Faculty of Health Sciences, San Jorge University, Villanueva de Gállego, Zaragoza, Spain; 3 Pharmaceutical Research Group of the University of Granada, Faculty of Pharmacy, Granada University, Granada, Spain; 4 Graduate School of Health, Discipline of Pharmacy, University of Technology Sydney, Sydney, NSW Australia

**Keywords:** Naturalistic, Comparative effectiveness research, Pragmatic clinical trials, Pharmaceutical care, Community pharmacy services, Clinical pharmacy services, Professional pharmacy services, Community pharmacy

## Abstract

**Background:**

Implementation of Professional Pharmacy Services (PPSs) requires a demonstration of the service’s impact (efficacy) and its effectiveness. Several systematic reviews and randomised controlled trials (RCT) have shown the efficacy of PPSs in patient’s outcomes in community pharmacy. There is, however, a need to determine the level of evidence on the effectiveness of PPSs in daily practice by means of pragmatic trials.

To identify and analyse pragmatic RCTs that measure the effectiveness of PPSs in clinical, economic and humanistic outcomes in the community pharmacy setting.

**Methods:**

A systematic search was undertaken in MEDLINE, EMBASE, the Cochrane Library and SCIELO. The search was performed on January 31, 2020. Papers were assessed against the following inclusion criteria (1) The intervention could be defined as a PPS; (2) Undertaken in a community pharmacy setting; (3) Was an original paper; (4) Reported quantitative measures of at least one health outcome indicator (ECHO model); (5) The design was considered as a pragmatic RCT, that is, it fulfilled 3 predefined attributes. External validity was analyzed with PRECIS- 2 tool.

**Results:**

The search strategy retrieved 1,587 papers. A total of 12 pragmatic RCTs assessing 5 different types of PPSs were included. Nine out of the 12 papers showed positive statistically significant differences in one or more of the primary outcomes (clinical, economic or humanistic) that could be associated with the following PPS: Smoking cessation, Dispensing/Adherence service, Independent prescribing and MTM. No paper reported on cost-effectiveness outcomes.

**Conclusions:**

There is limited available evidence on the effectiveness of community-based PPS. Pragmatic RCTs to evaluate clinical, humanistic and economic outcomes of PPS are needed.

**Supplementary Information:**

The online version contains supplementary material available at 10.1186/s12913-021-06150-8.

## Background

The pharmacy profession is constantly evolving from the traditional role of dispensing medicines towards patient-centred and collaborative care [1, 2], as it adapts to the demands and needs of patients [3, 4]. This practice change [5], internationally acknowledged and supported by professional organisations [6, 7], involves expanding the roles of pharmacists, by increasing their responsibility for the outcomes of medication therapy [8]. In community pharmacy, the change is operationalized through the implementation of professional pharmacy services (PPSs). A professional pharmacy service is defined as “*an action or set of actions undertaken in or organised by a pharmacy, delivered by a pharmacist or other health practitioner, who applies their specialized health knowledge personally or* via *an intermediary, with a patient/client, population or other health professional, to optimise the process of care, with the aim to improve health outcomes and the value of health care* [9]*.*”

In many countries PPSs are not integrated into daily practice due to a variety of factors. The main barriers are lack of evidence, lack of government support and/or no remuneration for service provision. However, the implementation process in various countries is at different phases and has followed different paths [10–12]. In some countries [12, 13] such as Australia, the United States of America (USA), Canada and the United Kingdom (UK), community pharmacists obtain reimbursement for providing these patient oriented services [2]. Most services are reimbursed by governments using a fee for service approach [14–17]. Implementation of reimbursed PPS normally requires a previous demonstration of the service’s impact (efficacy) and its effectiveness by means of high-quality research. However, translating these research findings into practice has been challenging [18, 19]. It is widely accepted that randomised control trials (RCTs) of various types are the appropriate research design to evaluate the efficacy of services while effectiveness should be assessed by means of pragmatic or naturalistic trials [20]. Generally, explanatory RCTs tend to maximize the accuracy of the results (internal validity) at the expense of external validity (the ability for a result to be applied or used in a particular situation) [21]. The term pragmatic is used for trials that test the effectiveness of the intervention in many clinical practice settings (e.g., inpatient hospitals, emergency departments) [11, 22–24] maximizing applicability and generalisability [25–27] (high external validity) of their results. Observational designs are also used to assess effectiveness [28, 29].

Several RCTs and systematic reviews have reported the clinical, economic and humanistic outcomes of PPSs in community pharmacy [30–41], although further research may be needed to determine whether these services can improve health-related quality of life and reduce healthcare costs [42]. Most of the studies reported in the literature have used an explanatory RCT design, which result in low external validity with consequent limitations associated with the generalisation of results [43–46]. Results from pragmatic trials may better meet the needs of stakeholders, healthcare professionals, payers [47–49] and patients [21]. As a result, there has been numerous calls to generate a high level of evidence on the clinical, economic and humanistic effectiveness of PPSs in daily practice by means of pragmatic trials [50–53]. It is, however, not easy, to differentiate between an explanatory and a pragmatic trial. Most trials seem to include both aspects, acknowledging the notion that explanatory and pragmatic exist in a continuum [54–57]. The PRECIS-2 tool was developed and validated to allocate research designs into this continuum. This tool consists of 9 domains scored from 1 (very explanatory) to 5 (very pragmatic). The domains are: eligibility, recruitment, setting, organisation, flexibility (delivery), flexibility (adherence), follow-up, primary outcome, and primary analysis. The main limitation of the PRECIS- 2 tool is that there may be interrater differences when applying each criterion. However, it provides explicit criteria that enhances discussion and eventual consensus [58, 59].The PRECIS-2 tool has shown a modest discriminatory validity [60].

This study aims to systematically review the literature to identify and analyse randomised controlled pragmatic trials that measure the effectiveness of the PPSs in the community pharmacy setting.

## Methods

A systematic search was undertaken in MEDLINE, EMBASE, the Cochrane Library and SCIELO, following PRISMA guidelines [61].

### Search strategy

For the search performed in MEDLINE/PubMed the following Mesh terms and key words were used:“Outcome Assessment (Health Care)”[Mesh] OR (pragmatic[All Fields] OR (naturalistic[All Fields])))) OR (“Comparative Effectiveness Research”[Mesh] OR “Pragmatic Clinical Trials as Topic”[Mesh] OR “Pragmatic Clinical Trial” [Publication Type]))) AND (“pharmaceutical care”[tiab] OR “medication review”[tiab] OR “Community Pharmacy Services”[Mesh] OR “Drug Utilization Review”[Mesh] OR “Medication Therapy Management”[Mesh] OR “clinical pharmacy service”[All Fields] OR “pharmacists”[MeSH Terms]). Filters: published in the last 10 years. The search strategies used in the other databases are included in Appendix. The search was performed by three of the authors (MAG, LSB and RVD) and the last update was made on January 31, 2020.

### Selection criteria

The following inclusion criteria were used for the selection process of papers: (1) The intervention assessed could be defined as a PPS according to Moullin’s definition [9]; (2) The intervention assessed was performed in the community pharmacy setting; (3) Was an original paper; (4) The trial reported quantitative measures of at least one health outcome indicator (ECHO model) [62]; (5) The study design was considered as a pragmatic RCT, that is, it fulfilled 3 of the 4 following key attributes [63, 64]: (a) analyzed the effectiveness of an intervention, (b) included more than 200 patients in each arm, (c) was undertaken in routine health care, (d) used broad eligibility criteria. Only articles in English and Spanish languages were included.

### Data collection and analysis

Duplicated records were removed using Endnote©. The selection process was undertaken by the same three authors (MAG, LSB and RVD). Two independent researchers (RVD, MAG) reviewed the literature; and disagreements were resolved with a third reviewer (LSB). In addition, a manual search was performed for references not identified in the search through the references list of the retrieved papers. Abstracts were screened and excluded if they did not meet the inclusion criteria. Abstracts with insufficient information were assessed in full text. After excluding at the abstract level, the complete text of the remaining references was assessed against the same selection criteria.

The data-extraction form, after being piloted with a sample of three papers, included:

- Degree of pragmatism of the studies. To further characterize the degree of pragmatism of the finally included papers, the PRECIS-2 tool was used [65]. Three authors (MAG, LSB and RVD) rated the degree of pragmatism, scoring independently each trial/study on each of the 9 domains of the PRECIS-2. After the independent assessment there were minor differences which were resolved through a moderation-consensus approach.
- Internal validity was analyzed following the Centre for Reviews and Dissemination’s guidance for undertaking reviews in health care. The following methodological aspects were selected to assess quality of the papers for this review; measure of variability, baseline comparability, contamination risk and blinded assessment [59].
- External validity was analyzed with the PRECIS- 2 tool [65].
- Description of general characteristics of included papers: type of PPS (interventions were categorized according to a hierarchical model [66], author, country, publication year, number of patients, duration, objectives, design/ method, patient inclusion/exclusion criteria and outcomes indicators).
- Description of the PPS components and summary of the clinical, economic or humanistic outcomes.

Trial registration: PROSPERO 2018 CRD42018073286 Available from:

https://www.crd.york.ac.uk/prospero/display_record.php?RecordID=73286

## Results

### Search description

**Fig. 1:**
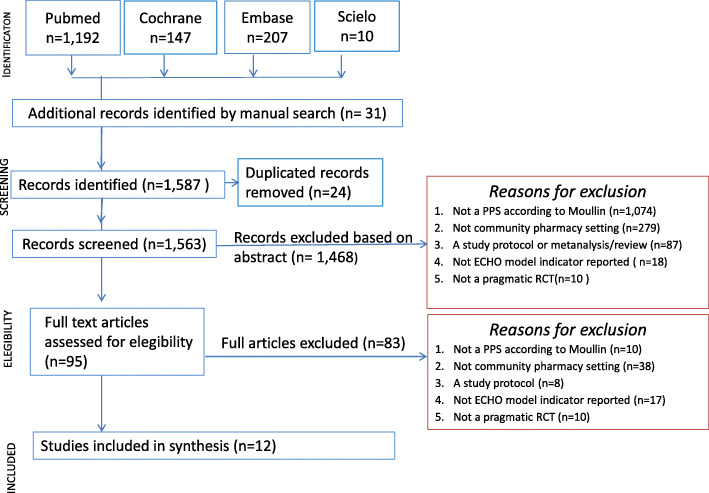
Flow diagram of the screened, assessed for eligibility, included and excluded papers according to PRISMA

The search strategy retrieved 1556 papers and 31 additional papers were identified from the manual search from references of the included papers. After removing duplicates, a total of 1563 references remained. During the screening of abstracts, 1468 articles were excluded. Ninety-five papers in full text were assessed, of which 83 were excluded because they did not meet the selection criteria. Finally (Fig. 1), a total of 12 pragmatic RCTs were included [49, 51, 67–76].

### Studies description

Of the 12 studies identified as pragmatic, three were cluster randomised controlled trials (cRCT) [49, 51, 72] while the other nine were RCTs [67–71, 73–76].The duration of the studies varied between three [49, 67, 72, 76] and 14 months [68] with a mean length of 6.4 months (SD = 3.6). The sample size varied from 65 [70] to 6987 patients [49]. The mean number of patients included in the studies was 1029.75 (SD = 1892.0) with a median of 541.0.

The mean number of pharmacists per study delivering the intervention was 55.2 (SD = 37.5) although in one paper only one pharmacist was involved [76]. In six of the studies pharmacists were reimbursed for providing PPS [51, 68–70, 72, 74]. In seven of the 12 studies there was training for the intervention [49, 51, 69–73], either face-to-face, on-line or both, with a duration of between six and 48 h. In the remaining five studies [67, 68, 74–76] no training was delivered as the pharmacies or pharmcists were already accredited for PPS provision.

#### Degree of pragmatism and study quality

**Fig. 2:**
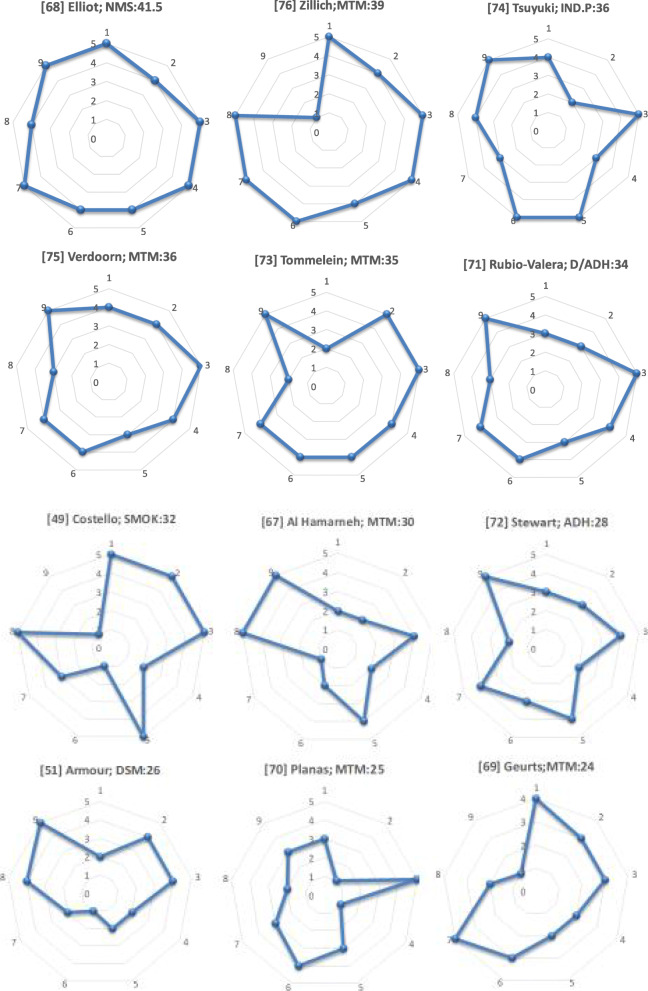
Assessment of pragmatic degree of trials using PRECIS-2 tool. Image shows PRECIS-2 wheels for PPS according to the degree of pragmatism: author; PPS: PRECIS-2 score out of 45. 1. Eligibility, 2. Recruitment, 3.Setting, 4.Organisation, 5. Flexibility (delivery), 6. Flexibility (adherence), 7. Follow-up, 8. Primary outcome, 9. Primary analysis

All eligible papers scored over 50% on the PRECIS-2 scale, which reinforced their external validity and were considered pragmatic. The scores ranged from 24.0/45 to 41.5/45 (Fig. 2). The highest value for each PRECIS-2 domain were obtained for Setting (average 4.6/5) and Primary analysis (average 3.8/5) while the lowest value was found in Organisation criteria (average 3.0/5). The complete assesesment of PRECIS-2 tool is available in the Additional file 1.

With respect to internal validity, nine papers stated confidence intervals or size effects for their main outcome indicators [49, 67, 68, 71–76]. Only three papers presented baseline differences between groups [70, 71, 75] and these used a statistical technique to adjust for such differences. In six out of the nine non cluster RCT, the authors recognized contamination risk between groups [68, 69, 71, 73–75]. Only one RCT used a blinded assessor [71]. Nine out of 12 analyses were based on intention to treat (ITT) [51, 67, 68, 70–75] whereas the remaining three were per protocol [49, 69, 76].

### Type of PPSs

The PPSs reported were classified as eight different types [66]: Dispensing/Adherence service [71, 72]; Smoking cessation service [49]; New Medicine Service (NMS) [68]; Independent prescribing [74]; Medication Therapy Management (MTM) [67, 70, 76]; Clinical Medication Reviews (CMR) [69, 75]; Disease State Management (DSM) [51] and Pharmaceutical Care (PC) [73]. Since CMR [69, 75], DSM [51], PC [73] and MTM services [67, 70, 76] included a care plan, implying a continuous intervention and evaluation of patients’ outcomes, they were recategorized as MTM [77]. Therefore, five different types of PPSs were finally evaluated.

Pharmacists delivered the service by means of an individualized face to face consultation with patients [49, 51, 69–75], telephone contact [68, 76], or review of patient’s records without patient contact [67].

Four out of the 12 studies [49, 51, 72, 73] reported patients’ loss to follow up rates higher than 15%. The main causes of dropouts were hospitalization [73], inability to contact [75, 76], not giving informed consent [75], lost to follow up [49, 51, 67, 70, 71, 73], death [73, 75], not having time for the study and for no specific reason [67, 68, 74].

Further descriptions of the general characteristics of included articles are provided in Table 1.

**Table 1:** Description of key general characteristics of included papers

Author / PPS/Country/ Publication year / Duration/ Number of patients	Objectives	Outcome indicators
[67] **Al Hamarneh** **MTM** Canada/2017/ 3 months *n* = 573; IG (*n* = 286) and CG (*n* = 287)	To evaluate the effect of pharmacist case finding and intervention program on estimated cardiovascular (CV) risk in patients with diabetes.	**Primary:** Difference and change in estimated CV risk (validated risk-assessment equation) between IG and CG. **Secondary** Differences between groups in changes of: HbA1c, LDL cholesterol levels, blood pressure and tobacco cessation, medication use and dose changes, lifestyle habits and CV risk and risk factors.
[51] **Armour** **DSM asthma/MTM** Australia/2013/ 6 months *n* = 570; IG (*n* = 292) and CG (*n* = 278)	To test the feasibility, effectiveness and sustainability of an asthma service.	**Primary:** Asthma control (validated assessment equation- ACQ-), inhaler technique and health - related quality of life.
[49] **Costello** **Smoking cessation** Canada/2010/3 months *n* = 6987; IG (Group A (*n* = 3588) and Group B (*n* = 3399))	To evaluate the effectiveness of two models of smoking cessation that included nicotine replacement therapy (NRT).	**Primary:** Abstinence at end-of-treatment determined by self-report.
[67] **Elliot** **NMS** England/2017/ 14 months *n* = 504; IG (*n* = 251) and CG (*n* = 253)	To examine the effectiveness of the NMS in people starting a new medicine for a long-term condition.	**Primary:** Adherence (validated assessment equation), health status (quality of life), medicines understanding and National Health System (NHS) cost.
[69] **Geurts** **CMR/MTM** Netherlands/2016/12 months *n* = 512; IG (*n* = 248) and CG (*n* = 264)	To determine whether a medication review followed by a pharmaceutical care plan decreases potential DRPs and pharmaceutical care issues (PCIs), along with a positive effect on cardiovascular risk factors and safety parameters for elderly polypharmacy patients with a cardiovascular disorder.	**Primary:** Resolved DRPs and PCIs. **Secondary:** Differences in clinical and lab values
[70] **Planas** **MTM** United States/2012/ 9 months *n* = 65; IG (*n* = 38) and CG (*n* = 27)	To evaluate the efficacy of a community- based, pharmacist-directed diabetes management program among managed care organization enrolees using National Committee for Quality Assurance (NCQA)–Healthcare Effectiveness Data and Information Set (HEDIS) performance measures.	**Primary:** HbA1c (< 7.0%), blood pressure (< 130/80 mmHg), and LDL cholesterol (< 100 mg/dL). A composite research outcome of success was created by determining whether a participant achieved two of the three goals at the end of 9 months.
[71] **Rubio-Valera** **Dispensing /Adherence service** Spain/2013/6 months *n* = 179; IG (*n* = 87) and CG (*n* = 92)	To evaluate the effectiveness of a community pharmacist intervention (CPI) compared with usual care in improvement of adherence to antidepressants and patient well-being in a population initiating pharmacological treatment following diagnosis of depression.	**Primary:** Adherence to antidepressants, clinical severity of depression, health-related quality of life and satisfaction with pharmacy care.
[72] **Stewart** **Adherence service** Australia/2014/ 6 months *n* = 395; IG (*n* = 207) and CG (*n* = 188)	To evaluate a community pharmacist intervention to improve adherence with antihypertensive medicines with a view to improving blood pressure (BP) control.	**Primary:** Change in proportion self-reporting medication adherence (validated questionnaire). **Secondary:** BP changes and the proportion of self-reporting adherence on the Tool for Adherence Behaviour Screening (TABS).
[73] **Tommelein** **PC/MTM** Belgium/2013/ 3 months *n* = 734; IG (*n* = 371), CG (*n* = 363)	To test the effectiveness of a pharmaceutical care programme in patients with Chronic Obstructive Pulmonary Disease (COPD) in the community pharmacist educational intervention.	**Primary:** Improvement of the inhalation technique, and medication adherence, **Secondary:** Reduction of Dyspnoea, severe exacerbations and emergency visits or hospitalization. Improvement of generic health status (quality of life) and disease- specific health status - COPD Assessment Test (CAT)-.
[74] **Tsuyuki** **Independent prescribing** Canada/2015/ 6 months *n* = 248; IG (*n* = 181), CG (*n* = 67)	To study the impact of pharmacist prescribing on blood pressure (BP) control in community-dwelling patients.	**Primary:** Difference in change of systolic BP from baseline to 6 months between IG and CG. **Secondary:** Change in diastolic BP. Number of: patients at their target BP after 6 months, new antihypertensive medication, dose changes, drug changes, new prescriptions for ASA and cholesterol lowering medications.
[75] **Verdoorn** **CMR/ MTM** Netherlands/2019/ 6 months *n* = 629; IG (*n* = 315) and CG (*n* = 314)	To investigate the effect of a patient -centred CMR, focused on personal goals, on health-related quality of life (HR-QoL), and on number of health problems.	**Primary:** HR-QoL (assessed with EuroQoL [EQ]-5D-5L and EQ-Visual Analogue Scale [VAS]) Number of health problems, after 3 and 6 months. **Secondary:** Number of: long-term medications, prescribed drugs added and ceased. Severity of complaints measured with VAS scores and healthcare consumption.
[76] **Zillich** **Telephone MTM** USA/2014/ 3 months *n* = 961; IG (*n* = 475) and CG (*n* = 486)	To evaluate the effectiveness of a telephonic MTM service on reducing hospitalizations among home health patients.	**Primary:** 60-day all-cause hospitalization. **Secondary:** The effect of hospitalization while adjusting for patients’ baseline risk. Number of medications taken daily. DRP identified. Pharmacist’s recommendations. Physician’s response.

#### Clinical, economic or humanistic outcomes of the interventions

Nine out of the 12 studies showed positive statistically significant differences in a clinical primary outcome that could be associated with four PPS: Smoking cessation [49], Dispensing/Adherence service [71], Independent prescribing [74] and MTM [51, 67, 69, 70, 73, 76]. The other three studies only achieved significant statistical improvements in subgroup analysis [75], secondary outcomes [72] and/or process indicators [68]. Description of the interventions and detailed clinical, economic or humanistic primary outcomes of papers included are provided in Table 2.

#### Clinical outcomes

Provision of specific PPSs achieved a statistically significant better control of several clinical outcome indicators: Independent prescribing improved blood pressure [74], Adherence service improved blood pressure [72], Smoking cessation improved abstinence from tobacco [49] and MTM improved pulmonary exacerbations [73], HbA1c [67, 70], CV risk [67], blood pressure [67, 69, 70, 72, 74], tobacco use [67], HDL [69] and LDL-cholesterol [67]. Additionally, eight papers showed a statistically significant improvement in process indicators of PPSs effectiveness, such as the inhaler technique [51, 73], adherence [51, 68, 71–73] or resolution of drug related problems (DRPs) [69, 75, 76]. MTM statistically decreased hospitalizations, bed-bound episodes and emergency room visits [67, 71, 73, 74, 76].

#### Humanistic outcomes

Three out of the five papers [51, 68, 71, 73, 75] measuring humanistic outcomes reported statistically significant improvements in Health-Related Quality of Life (HR-QoL) through the provision of Dispensing/ Adherence [71] and MTM services [51, 75]. In these three papers, HR-QoL was the primary outcome.

#### Economic outcomes

None of the papers involved any cost-effectiveness analysis. NMS [68] didn’t show a statistically significant reduction in National Health Service (NHS) costs.

**Table 2:** Description of the interventions and detailed clinical, economic or humanistic primary outcomes of papers included

Author PPS	PPSs components	Health outcomes
[67] Al Hamarneh MTM	Assessment of patient’s therapies and laboratory results, individualized CV risk assessment, adjustment of treatment regimen, prescribing and ordering laboratory tests to meet treatment targets, and self-report of adverse events. Regular communication after each contact with the patients and regular follow-up visits every 4 weeks for 3 months.	Improvement of: CV risk (absolute reduction 5.38%; 95%CI, 4.24–6.52; *P* < 0.001), (HbA1c (0.9%; 95%CI, 0.70–1.10; *P* < 0.001), systolic blood pressure (8.6 mmHg; 95%CI, 6.70–10.40; *P* < 0.001), diastolic blood pressure (2.7 mmHg; 95%CI, 1.30–4.10; *P* < 0.001), LDL-cholesterol, (0.2 mmol/L; 95%CI, 0.10–0.30; *P* = 0.004) and Tobacco use (24.2% *P* < 0.001).
[51] Armour DSM/MTM	Brief health education tips. Inhalation technical instruction. Letter written to the doctor for the action plan. Asthma control and spirometry performed at every visit measured using questionnaires and spirometers.	Improvement of: percentage of patients achieving a good/fair control in both groups 4-visit service: from 21 to 59%; 3-visit service: from 29 to 61% (mean = 0.57 for the three-visit group, 0.56 for the four-visit group, *P* < .001). Improvement of the health-related quality of life (Three visit 4.13 ± 1.41 to 3.39 ± 1.19; *P* < .001- versus Four-visit 4.45 ± 1.49 to 3.57 ± 1.48; *P* < .001). No significant differences of asthma control between patients receiving 4 visits compared to patients receiving 3 visits.
[49] Costello Smoking cessation	Pharmacist – led behavioural counselling combined with nicotine replacement therapy (NRT). 1 (Group B) to 3 (Group A) face-to-face sessions for behaviour change to quit smoking. On-line control surveys at 7 days, and 5 and 12 weeks. Phone calls to those who did not attend the visits or respond.	There were statistically significant differences between each of the two groups receiving the service and the group of patients that only received NTR by mail (control group) 3-session service (× 2 = 217.30, P < 0.001; ITT: × 2 = 149.60, *P* < 0.001); 1-session service (× 2 = 93.90, *P* < 0.001; ITT: × 2 = 19.00, *P* < 0.001).
[68] Elliot NMS	Pharmacist and GP service offering to the patient. The pharmacist asks about adherence and medicines. One-to-one consultation 7–14 days after the presentation of the prescription with a ‘follow-up’ of 14–21 days via telephone.	Improvement of the percentage of adherent patients 1.67 (95%CI, 1.06–2.62; *P* = 0.027). Non-significant reductions of health system costs (£21; 95%CI, £59 - £150; *P* = 0.1281).
[69] Geurts MTM	Pharmaceutical care Process (PCP) in cooperation between patient’s pharmacist and GP, and agreed to by the patient: (1) assessment of potential DRPs and pharmaceutical care issues (PCIs), (2) proposal of interventions to reach treatment goals, and (3) implementation of the interventions. Two measurements were performed, (t = 0) at the beginning and (t = 1) after 1-year follow up.	Decrease of diastolic BP (95%CI, 79.80–76.80 mmHg; *P* = 0.008) and increase of HDL-cholesterol: IG (IG: 95%CI, 1.29–1.37 mmol/L; *P* = 0.021; IG patients not receiving the whole service: 95%CI, 1.26–1.37 mmol/L; *P* = 0.039); and GC: (95%CI, 1.30–1.36 mmol/L; *P* = 0.074). Non-significant decrease of LDL-cholesterol: IG (IG: 95%CI, 2.72–2.63 mmol/L; *P* = 0.337; IG patients not receiving the whole service: 95%CI, 2.98–2.67 mmol/L; *P* = 0.740); and CG: (95%CI, 2.61–2.58 mmol/L; *P* = 0.032).
[70] Planas MTM	1-h face to face interview on a monthly basis (IG) and 30 min face to face interview at 3- month intervals (CG). IG: 1) Provision of written patient education materials. 2) Diabetes education, coaching on self-management skills and medication adherence. 3) Assessment of medications and DRP. 4) Contact with GP via fax or telephone to recommend treatment adjustments.	Improvement of the percentage of patients achieving the control of their health problem: HbA1c (IG: 46.70% vs. CG: 9.10%, *P* < 0.002), blood pressure (IG: 53.30% vs. CG: 22.70%, *P* < 0.020). Non-statistically significant higher percentage of patients achieving the LDL target levels (IG: 50.00% vs. CG: 46.70%, *P* = 0.460).
[71] Rubio-Valera Dispensing/ Adherence service	First visit: educational intervention centred on improving patients’ knowledge of antidepressants and awareness of the importance of adherence and quality of life. Subsequent visits: short review of some points covered in the first visit and checking of patient progress.	Improvement of the health-related quality of life (0.25 vs. 0.14) - effect size 0.31 vs 0.33 -. No statistically significant differences in adherence, satisfaction or clinical severity depression.
[72] Stewart Adherence service	Home BP monitor. Training on BP self-monitoring (3–6-month follow-ups). Motivational interviewing and education to medication adherence. Medication use review. Referral to a GP Prescription refill reminders.	Improvement of the proportion of adherent patients although there were not significant differences between groups (57.2 to 63.6% CG vs 60.0 to 73.5% IG, *P* = 0.23). Reduction of systolic BP was significantly greater in the IG (7.2 mmHg 95%CI 1.6 12.8 mmHg; *P* = 0.001). Reductions in BP of 10.00 mmHg (IG) vs. 4.60 mmHg (CG); *P* = 0.050. Improvement of percentage of non-adherent patients becoming adherent 22.60% (95%CI, 5.10–40.00%) in the IG compared to CG (IG: 61.80% vs. CG: 39.20%; *P* = 0.007).
[73] Tommelein MTM	Educational intervention (two sessions of 15–25 min). Electronically recorded medication, inhalation technique and questionnaires about behavioural issues, etc. Letter to the patient’s GP.	Significantly lower estimated annual severe exacerbation rate in the IG compared with the CG (0.27 (IG) vs. 0.61 (IC): RR = 0.45; 95%CI, 0.25–0.80; *P* < 0.007). Also, significantly 72% lower estimated annual hospitalization rate in IG vs CG (0.10 vs. 0.40; RR = 0.28; 95% CI, 0.12–0.64; *P* = 0.003) and a statistically significant 73% lower rate of hospitalization days (0.87 vs. 3.51; RR, 0.27; 95%CI, 0.21–0.35; *P* < 0.0001).
74] Tsuyuki Independent prescribing	Assessment of BP and cardiovascular risk. Education on arterial hypertension. Prescribing of antihypertensive medications. Laboratory monitoring and monthly follow-up visits for 6 months. Provision of a wallet card for BP recording.	Greater reduction of systolic BP in the IG of 6.60 (1.90) mmHg (*P* = 0.0006) and proportion of patients achieving target BP 58% (IG) vs. 37% (CG), *P* = 0.020); OR = 2.32 (95%CI, 1.17–4.15).
[75] Verdoorn CMR/MTM	First visit: a patient interview for gathering information (health problems, preferences, and all medications used). Identify potential DRPs and propose recommendations to solve them. Subsequent visits: face-to-face meeting with the patient’s GP to discuss all health-related goals and DRPs. Two follow up appointments.	Improvement of the health-related quality of life: 3 months 1.70 points (95% CI, 0.47–2.90; *P* = 0.006) and 6 months 3.40 points (95% CI, 0.94–5.80; *P* = 0.006).
[76] Zillich Telephone MTM	Home episodic skilled nursing care. Medication information was faxed from nurse to the provider. Initial phone call by a pharmacy technician to verify active drugs. Pharmacist-provided telephone MTM. Follow - up pharmacist phone calls at 7-day and as needed for 30 day of the 60-day home health care episodes.	Significant less probability of hospital readmission in patients with a low baseline risk (adjusted OR: 3.79; 95%CI, 1.35–10.57; *P* = 0.01). No significant differences in the 60- day probability of hospitalizations adjusted OR: 1.26; (95%CI, 0.89–1.77; *P* = 0.190).

## Discussion

Stakeholders need pragmatic studies with high methodological quality to make evidenced based decisions on which services to fund. To the best of our knowledge, our paper is the first systematic review that evaluates evidence from pragmatic RCTs of the effectiveness of PPSs in the community pharmacy setting. One of the main strengths of this review is the use of the PRECIS-2 tool, which allowed the characterization of the level of pragmatism of the study designs. The PRECIS-2 tool had previously shown good reliability with modest discriminatory validity [60, 65]. Currently there are few pragmatic papers on PPSs and researchers should make wider use of this tool when designing their trials in an attempt to provide practice-based data on specific PPSs.

Setting was one of the highest scored domains within the studies, reflecting the ease of accessibility of community pharmacies to implement PPSs. The second most scored domain was the manner in which primary outcomes were assessed. Most of the studies used ITT which reinforced the external validity of their conclusions.

In contrast, the low score obtained in domains related to organisation criteria, recruitment and flexibility (adherence) suggests a need to implement strategies that enhance pharmacist training such as accreditation processes, training in communication skills and teaching of pragmatic methodologies [78–81]. According to our assessment, studies with the highest score of pragmatism were carried out in UK [68], Canada [74], Netherlands [75] and US [76], countries coinciding with those described by Mossialos et al. [2], as the most advanced PPSs providers. Probably these countries provide a more favorable environment for patient recruitment and PPSs implementation, with experience in service accreditation and remuneration.

Our findings reinforce previous conclusions on the effectiveness of PPSs [30, 82]. There was evidence (nine out of the 12 pragmatic RCT) that supports PPSs clinical effectiveness, specifically for prescribing interventions and MTM services. These two services achieved statistically significant improvements in a variety of intermediate clinical outcomes such as blood pressure, pulmonary exacerbations, HbA1c, CV risk, tobacco use, HDL and LDL- cholesterol. Other type of services (Dispensing /Adherence and NMS) did not report clinical effectiveness. The inherent methodologic components of the services, as well as other factors such as fidelity and duration of the intervention, may be related to favourable clinical outcomes. In Independent prescribing and MTM services, pharmacists assess patient clinical outcomes and develop interventions specifically tailored to individual patient’s health status. In contrast, pharmacists providing Dispensing/Adherence and NMS services focus their interventions predominantly on improving the medication use process (inhaler technique, DRP).

The two studies with the highest pragmatic scores, one evaluating a telephonic MTM service and the other assessing the NMS, did not demonstrate any positive results on hospitalizations nor medication adherence. This may be due, or related to, the short duration of the studies (3 months and 10 weeks respectively) or to the components of the service (i.g. telephonic follow-up). The additional components or factors that may make telephonic MTM or follow-up effective are unknown [83, 84].

There were three pragmatic studies supporting the effectiveness of PPSs (asthma DSM, Dispensing/Adherence and MTM) on humanistic indicators (HR-QoL) [51, 71, 75]. The studies that achieved statistically significant improvements had follow-up visits and assessed the effect at 6 months. These two components had been previously reported as critical in achieving a humanistic impact [34, 42, 82]. Therefore, studies measuring humanistic indicators should consider longer durations and follow-up periods.

Although there are many systematic reviews of PPSs cost-effectiveness, these are undertaken in explanatory RCTs [35, 36, 85]. In our review none of the papers fulfilling our selection criteria for pragmatism assessed cost-effectiveness. Three articles [68, 73, 76] reported other economic indicators but in none of these there was any significant statistical difference between groups. The importance to stakeholders of cost-effectiveness studies should not be underestimated since the sustainability of services might be dependent on delivering costs savings or cost-effectiveness to the healthcare system.

Two key methodological quality aspects should be considered when designing future pragmatic research. We found only one study that blinded outcome assessment, which may be considered as a potential bias that could overestimate PPS effectiveness. Half of the studies reported a risk of contamination, a very common threat in the assessment of complex interventions. Thus cluster designs controlling for potential risk of contamination should be considered.

This review has several limitations due to the assessment of pragmatism.The PRECIS-2 tool was applied after the screening process which allowed only the consideration of papers meeting our inclusion criteria. Since the PRECIS-2 tool suggests a continuum between explanatory and pragmatic and no minimum score is provided to classify a paper as pragmatic or explanatory, the selected cut-off point (22.5 out of 45) could be questioned.The PRECIS-2 tool although having good reliability has been reported to have modest discriminatort validity [60]. Another limitation relates to the variability of the different PPSs type and their outcomes making it difficult to make comparisons between studies. Future studies should select specific services to evaluate health outcomes using pragmatic approaches, using the PRECIS-2 tool to design the study.

## Conclusions

There is a need for pragmatic studies to evaluate economic, clinical and humanistic outcomes of PPS as currently there is limited available evidence on the effectiveness of these services.

Although few pragmatic RCT were found, most of them showed evidence of clinical effectiveness. There is scarce evidence of humanistic and economic outcomes. The lack of an adequate number of pragmatic clinical trials may be problematic to provide evidence of the sustainability of the PPS. More focus and emphasis should be given to increase the level of evidence of economic, clinical and humanistic indicators from a pragmatic perspective.

Researchers should consider the level of pragmatism of their research design using the PRECIS-2 tool. This would allow them to ensure the applicability of their research.

### Practical implications

There is limited evidence of PPS outcomes from pragmatic studies. Therefore there is a need to undertake further research on specific PPS, with patients under normal practice conditions.

## Supplementary Information

**Additional file 1.** The complete assessment of pragmatic degree of trials using of PRECIS 2-tool

## Data Availability

The datasets used and/or analysed during the current study are available from the corresponding author on reasonable request.

## Appendix

Search strategy used in the other databases.

**COCHRANE:**

((pragmatic[All Fields] OR (naturalistic[All Fields]) OR (“Comparative Effectiveness Research”[Mesh] OR “Pragmatic Clinical Trials as Topic”[Mesh]) AND (“pharmaceutical care”[tiab] OR “medication review”[tiab] OR “Community Pharmacy Services”[Mesh] OR “Drug Utilization Review”[Mesh] OR “Medication Therapy Management”[Mesh] OR “clinical pharmacy service”[All Fields] OR “pharmacists”[MeSH Terms])

**EMBASE:**

(pragmatic OR naturalistic) AND (**Pharmaceutical Care** OR **medication therapy management** OR **clinical pharmacy** OR **pharmacists** OR Community Pharmacy or Drug Utilization Review)*.

* Those in bold are Embase Emtree terms.

**SCIELO:**

(Pragmático or naturalistico or efectividad) AND (servicios profesionales OR atención farmacéutica OR seguimiento farmacoterapéutico OR revisión de la farmacoterapia OR farmacéutico OR farmacia comunitaria OR gestión de la medicación OR revisión del uso del medicamento)

## Abbreviations

ACQ: Asthma Control Questionnaire
ADH: Adherence service
CAT: COPD Assessment Test
CMR: Clinical Medication Reviews
CV: Cardiovascular
D/ADH: Dipensing/Adherence service
DRP: Drug Related Problem
DSM: Disease State Management
ECHO: Economic, Clinic and Humanistic outcomes
HEDIS: Healthcare Effectiveness Data and Information Set
HR-QoL: EuroQoL [EQ]-5D-5L
MTM: Medication Therapy Management
NCQA: National Committee for Quality Assurance
IND.P: Independent prescribing
NHS: National Health System
NMS: New Medicine Service
NRT: Nicotine Replacement Therapy
PC: Pharmaceutical Care
PCI: Pharmaceutical care issues
PPS: Professional Pharmacy Service
RCT: Randomised Control Trial
SMOK: Smoking cessation service
TABS: Tool for Adherence Behavior Screening
VAS: EQ-Visual Analogic Scale
