# Supplementary material for: Systematic review of pragmatic randomised control trials assessing the effectiveness of professional pharmacy services in community pharmacies
Source: BMC Health Serv Res. 2021 Feb 17;21:156. doi: 10.1186/s12913-021-06150-8 (PMC7890900; doi:10.1186/s12913-021-06150-8)
Supplement: Supplementary file 1 — Additional file 1. The complete assessment of pragmatic degree of trials using of PRECIS 2-tool [file 12913_2021_6150_MOESM1_ESM.pdf]

ADDITIONAL FILE 1- The complete assessment of pragmatic degree of trials using of PRECIS 2-tool

	1.Eligibility	2.Recruitment	3.Setting	4.Organisation	5.Flexibility (delivery)	6.Flexibility (adherence)	7.Follow -up	8.Primary outcome	9.Primary analysis	Total/45	Average
[68] Elliot	5	4	5	5	4.5	4	5	4	5	41.5	4,55
[76] Zillich	5	4	5	5	4	5	5	5	1	39	4,33
[74] Tsuyuki	4	2	5	3	5	5	3	4	5	36	4,00
[75] Verdoorn	4	4	5	4	3	4	4	3	5	36	4,00
[73] Tommelein	2	5	5	4	4	4	4	2	5	35	3,88
[71] Rubio-Valera	3	3	5	4	3	4	4	3	5	34	3,77
[49] Costello	5	5	5	2	5	1	3	5	1	32	3,55
[67] Al Hamarneh	3	3	4	2	4	3	4	2	5	30	3,33
[72] Stewart	2	2	4	2	4	2	1	5	5	28	3,00
[51] Armour	2	4	4	2	2	1	2	4	5	26	2,88
[70] Planas	3	1	5	1	3	4	3	2	3	25	2,77
[69] Geurts	4	3	3	2	2	3	4	2	1	24	2,66
Total/45	42	40	55	36	43.5	40	42	41	46		
Average	3,5	3,33	4,58	3,00	3,58	3,33	3,50	3,41	3,83		
